# Does Air Abrasion of Dentin Affect the Bonding Performance of Different Universal Adhesives? 

**DOI:** 10.30476/dentjods.2025.103036.2413

**Published:** 2026-03-01

**Authors:** Fatemeh Safaei-Firoozabadi, Fatemeh Hashemi Moghadam, Seyedeh Farnaz Tabatabaei, Saeed Nemati, Farnaz Madisiar

**Affiliations:** 1 Dept. of Operative Dentistry, Faculty of Dentistry, Tehran Medical Sciences, Islamic Azad University, Tehran, Iran.

**Keywords:** Dental Air Abrasion, Tensile Strengths, Dental Leakage, Universal adhesive

## Abstract

**Background:**

Despite significant advancements in adhesive systems, the bond between tooth-colored restorations and dental hard tissues remains a challenge.

**Purpose:**

This study aims to evaluate the effects of air abrasion on the bond strength and microleakage of universal adhesives.

**Materials and Method:**

Eighty intact third molars were used in this *in vitro* study. Microtensile bond strength (μTBS) was tested on 32 teeth, and microleakage was assessed on 48 teeth. For μTBS testing, occlusal enamel was removed to expose a flat dentin surface. Standardized Class V cavities were prepared on the buccal and lingual surfaces for the microleakage test. Teeth were randomly divided into four groups according to the universal adhesive system used: All-Bond Universal, G-Premio Bond, G2-Bond Universal, and Clearfil SE Universal Bond. Each group was further split into two subgroups based on whether air abrasion pretreatment was applied. A 4-mm composite resin block (Tetric-N-Ceram, Ivoclar Vivadent) was built on the bonding surface for μTBS, and Class V cavities were restored with the same composite for microleakage evaluation. Then μTBS was measured using a universal testing machine, while microleakage was assessed via dye penetration. Data were analyzed using Tukey and t-tests for μTBS, and the Kruskal–Wallis test for microleakage, with significance set at α = 0.05.

**Results:**

Air abrasion significantly improved μTBS for All-Bond Universal and Clearfil SE Universal Bond (*p*< 0.001), G-Premio Bond (*p*= 0.041), and G2-Bond Universal (*p*= 0.027). However, it did not significantly affect microleakage (*p*= 0.32).

**Conclusion:**

Pretreating dentin with air abrasion enhances the bond strength of universal adhesives without increasing microleakage, supporting its use in restorative procedures.

## Introduction

Despite significant advancements in adhesive systems, tooth-colored restorations still face challenges at the bonded interface between dental hard tissues and resin-based materials [ 1 ].

Historically, poor marginal adaptation and loss of retention have been the most commonly reported causes of failure in adhesive restorations [ 2 ]. Achieving reliable adhesion at the interface between the dental substrate and the restoration is considered a critical factor influencing the longevity of restorations [ 3 ]. Therefore, dentin adhesives play a vital role in ensuring optimal marginal sealing of resin composites. Inadequate adaptation can lead to microleakage, which in turn may cause secondary caries, tooth sensitivity, pulp inflammation, and ultimately restoration failure [ 4 ].

This persistent challenge is largely due to the complexity of bonding to dentin, which arises from factors such as the fluid pressure within dentinal tubules, the high organic content of dentin, and the presence of the smear layer [ 5 ]. Improving long-term clinical outcomes often involves modifying the dentin surface to enhance resin bonding. Current adhesive strategies focus on the interaction between dental adhesives and the smear layer, aiming to achieve intimate adaptation to the tooth structure [ 6 ].

During cavity preparation, a smear layer is formed over the cut dentin surface, acting as a physical barrier. This layer must be either removed or rendered permeable to allow adhesive monomers to directly interact with the dentin. Various methods can be used to modify the dentin surface, resulting in smear layers with different characteristics [ 7 - 8 ]. The effectiveness of self-etch adhesives is influenced by the properties of this smear layer, which are affected by different dentin pretreatment techniques and, consequently, lead to varied bonding outcomes [ 9 ].

Air abrasion (AA) is one such cavity pretreatment technique that utilizes a fine stream of aluminum oxide particles propelled by compressed air. As the particles collide with the dentin surface, their kinetic energy causes microscopic fragmentation [ 10 ]. This results in a roughened tooth surface, which is more favorable for adhesive bonding.

The most recent advancement in adhesive technology is the development of universal adhesives, which adhere to the all-in-one approach, combining etching, priming, and bonding steps in a single bottle [ 11 - 12 ]. Among adhesive strategies, self-etch systems have emerged as the most consistent approach for bonding to dentin. These systems typically employ functional monomers, such as 10-methacryloyloxydecyl dihydrogen phosphate (10-MDP), which possess mild acidity and the ability to form stable, water-insoluble salts with calcium in the dentin [ 8, 13 - 14 ]. This chemical interaction is a primary reason why 10-MDP is widely included as the key adhesive monomer in most universal adhesives.

Universal adhesives represent a new direction in dental adhesion, driven by the need to simplify clinical procedures and reduce technique sensitivity [ 15 ]. Consequently, evaluating their bonding performance is of great importance. Although some studies have examined the bond strength of self-etch adhesives following air-particle abrasion pretreatment, comparative assessments among different universal adhesives remain limited [ 16 ]. D’Amario *et al*. [ 17 ] demonstrated that incorporating an additional AA step significantly enhanced the shear bond strength of total-etch adhesives.

The aim of the current work was to assess the impacts of AA pretreatment on dentinal surface on microleakage and bond strength of commercially accessible universal adhesives. The null hypothesis was the pretreatment of the dentin surface with AA does not significantly affect bond durability or microleakage scores compared to no pretreatment. 

## Materials and Method

This *in vitro* experimental study was conducted on human maxillary and mandibular third molars to evaluate the bond strength of four universal adhesive systems: All-Bond Universal (AL), G-Premio Bond (GP), G2 Bond Universal (G2), and Clearfil SE Universal Bond (CSE). Only extracted human third molars free from cracks, caries, restorations, or root canal treatment were included. Teeth exhibiting any form of restoration, decay, fracture, or previous endodontic treatment were excluded. Additionally, after sample preparation and sectioning, any specimens showing signs of bubbling or adhesive failure were eliminated from further testing.

The selected teeth were randomly assigned to four experimental groups (n= 8), based on the adhesive system used. Each group was further subdivided into two subgroups (n= 4), depending on whether AA pretreatment was applied.

Sample size estimation was performed using the one-way ANOVA power analysis function of PASS II software, indicating that an average standard deviation of ΔE= 0.85, with α= 0.05 and β= 0.1 (power= 90%), corresponded to an effect size of 0.58. Based on this analysis, a sample size of 12 was determined for each group. A purposive sampling method was employed.

Based on the fixed effects ANOVA power analysis option of PASS II software, with α= 0.05, the effect size on the adhesive variable with 4 levels is 1.08 and for the AA variable with 2 levels is 0.42. Taking 8 samples as the sample size of each subgroup, the statistical power on both variables is more than 0.99.

The experiment was performed using 32 intact human third molar teeth, with no restorations or caries lesions. Thoroughly, the teeth were cleaned after extraction, utilizing curettes and brushes and kept in 0.5% chloramine solution for 24h at room temperature. Then, the teeth were separated from the disinfectant solution, abundantly washed, kept in distilled water of the same temperature, and utilized within a month after extraction.

The teeth root portion was removed. To remove the whole occlusal enamel, it was sectioned with a circular diamond blade in an Isomet 1000 saw (Shenzhen Dian Fong Abrasives Co. China), with a speed of 150-200 RPM while cooling with water continuously to acquire flat dentin surface. The smear layer was standardized by polishing dentinal surfaces under running water with #600 grit SiC paper for 60s, rinsed for 15s and blot dried.

For the microleakage test, 48 intact human third molars, free of caries and restorations, were used. Following extraction, the teeth were meticulously cleaned using curettes and brushes to remove any debris. They were then stored in a 0.5% chloramine-T solution at room temperature for 24 hours for disinfection. Afterward, the teeth were thoroughly rinsed with distilled water and subsequently stored in distilled water at room temperature. All specimens were used within one month of extraction to ensure specimen integrity.

Class V preparations were performed on the lingual and buccal surfaces of each tooth with high-speed handpiece utilizing a diamond bur (Diatech, Heerbrurgg, Switzerland) while water-cooling. Gingival margin was placed on dentinal surface, 0.5 mm under the cemento-enamel junction (CEJ). Standardized preparations were obtained with the approximate width of 3mm (mesiodistal), depth of 1.5mm, and height of 2mm (occlusogingival) parallel to the CEJ. The bur was discarded after preparing every five cavities. A marked periodontal probe was used to verify dimensions of the preparations (Hu-Friedy Co.). The sample were assigned randomly into four classes based on the universal adhesives (n= 12). Then, each group was classified into two subclasses (n=6) in terms of with pretreatment AA.

The dentin was abraded with aluminum oxide particles (50μm), in the groups receiving surface treatments, with an angle of 90° between the dentin and jet, for 5 s, at a pressure of 60 PSI and a distance of 10 mm, utilizing a Micro Jato jet (Bio-Art; São Carlos, SP, Brazil). Then, the dentinal surface was rinsed for 15 s and dried with absorbent paper.

The adhesive systems were applied based on the manufactures instructions (Table 1). All adhesive systems were according to self-etch mode and polymerized with a Bluephase LED light (Ivoclar Vivadent AG, Schaan Liechtenstein, 800mW/cm^2^) for 20s. 

**Table 1:** The classification and composition of the universal adhesives tested

Application mode	Composition	Manufacturer	Adhesive
1. Apply two separate coats of adhesive, scrubbing the preparation with a micro brush for 10-15 s per coat.	MDP, Bis-GMA, ethanol	Bisco, Schaumburg, IL, USA	All-bond universal (AL)
2. Evaporate excess solvent by thoroughly air drying with an air syringe for at least 10 s; no Visible movement of the material was observed. The surface should have a uniform glossy appearance.			
3. Light cure for 10 s			
1. Apply using a micro brush.	10-MDP, 4-META, 10-methacryoyloxydecyl dihydrogen thiophosphate, methacrylate acid ester, distilled water, acetone, photo-initiators, silica fine powder	GC Corporation, Tokyo, Japan	G-Premio Bond (GP)
2. Leave undisturbed for 10 s.			
3. Dry thoroughly with air under maximum air pressure.			
4. Light cure for 10 s			
1. Apply primer to the entire dentin surface and leave for 10 s.	Primer: 4 MET 10-MDP MDTP Dimethacrylates, water, acetone, photoinitiator, filler Bonding agent: dimethacrylates, filler, photo initiator	GC, Tokyo, Japan	G2-bond universal(G2)
2. Air dry strongly for 5 s			
3. Apply bonding agent, air blow gently for 3 s			
4. Light cure for 10 s			
1. Apply bond to the entire cavity with a micro brush and rub it in for 10 s.	Bis-GMA, HEMA, ethanol, 10-MDP, hydrophilic aliphatic dimethacrylate, colloidal silica, DL camphorquinone, silane coupling agent, accelerators, initiators, water	Kuraray Noritake Dental Inc. Chiyoda Ku, Tokyo, Japan	Clearfill SE universal bond (CSE)
2. Dry with mild air stream for 5 s until the adhesive not move.			
3. Light cure for 10 s			

Abbreviations: 10-MDP= 10-methacryloyloxydecyl dihydrogen phosphate; Bis-GMA= bisphenol A-glycidyl methacrylate.; 4-META= 4-methacryloyloxyethy trimellitate anhydride; HEMA = 2-hydroxyethyl-methacrylate

After adhesive application, a composite resin block, Tetric-N-Ceram (Ivoclar Vivadent, Schaan Lichten stein) was built up on the bonding surface in the shade A2, in 4mm height, utilizing the material layers with the thickness of less than 1mm, each one cured with a Bluephase LED light (Ivoclar Vivadent AG, Schaan Liechtenstein, 800mW/cm^2^) for 20 s.

All samples were restored with Tetric-N-Ceram (Ivoclar Vivadent, Schaan Lichtenstein) after adhesive application, in the shade A2 and cured for 40 s using the same LED-curing unit. To polish restorations, PoGo micro-polisher point was used (Dentsply Caulk, USA) for 20 s.

All samples were kept in distilled water at 37∘C for 24 h. All groups were then exposed to 10,000 thermocycles (5-55C, transfer time: 5s, dwell time: 25s) (MTE 101 Thermocycling Machine, Esetron, Turkey).

The bonded teeth were implanted into acrylic resin (Acropars, Marlic, Iran) and were longitudinally cross sectioned with a diamond blade in Isomet 1000 saw (Shenzhen Dian Fong Abrasives Co., China), (speed of 150–200 rpm) while water cooling continuously. Thus, multiple beam-shaped sticks were obtained with a cross-sectional top of approximately 1 mm2. At least two stick specimens were obtained from each tooth (n= 8). The samples were studied through a Discovery V20 stereomicroscope (Binocular Motic SMZ-168, China). Thus, the sticks with adhesive failures and bubble inclusions were omitted.

The μTBS was examined with a universal testing machine (EMIC; São José dos Pinhais, PR, Brazil). Each beam ends were glued with cyanoacrylate adhesive to designed metal plates specially. Each beam was located in the testing machine. The tensile load was used at a crosshead speed of 0.5 mm/min, until separating the composite from the dentin. The load was recorded at the point of failure.

The adhesive failure (fracture at the interface between the dentin and resin) was defined along with cohesive (fracture within the body of the dentin or resin), or mixed (adhesive fracture integrated with cohesive fracture). Then, the samples were assessed by Discovery V20 stereomicroscopy (Binocular Motic SMZ-168, China) with 40× magnification.

**Figure 1:**
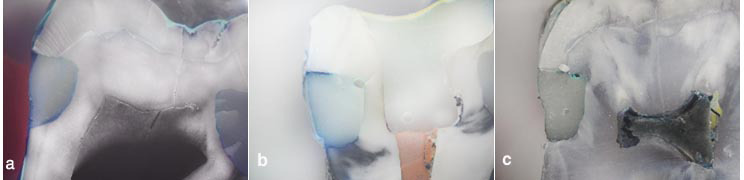
Light microscopy images of dye penetration at gingival margins at ×40 magnification, **a:** score 0, **b:** score 3, **c:** score 4

After thermocycling, the samples root apex was protected with a resin composite. Two layers of nail lacquer (Golden Rose, Turkey) were applied to cover the entire external surface of the teeth, leaving a 1mm margin around the restoration uncovered. Then, the specimens were submerged in 2% methylene blue while incubating for 24h at 37c. The samples were rinsed with tap water and classified longitudinally into two sections in a buccolingual direction with a slower speed saw (Isomet 4000, Buehler). The distal and mesial sections were assessed for leakage under light microscopy (Olympus Light Microscope, Japan) at 40× magnification by an inspector blinded to the experimental measures (Figure 1). 

To determine the microleakage score, the dye penetration depth was determined at the gingival margins based on ISO/TS,11405: 2003 [ 18 ] as (0) for no dye penetration, (1) for dye penetration to 1/2 of the gingival floor depth, (2) for dye penetration exceeding 1/2 of the gingival floor depth but not reaching the axial wall, (3) for dye penetration to the axial wall not including the wall and (4) for dye penetration including the axial wall.

To analyze all data, IBM SPSS Statistics V.26 statistical package was used (SPSS, Chicago, IL, USA). Using two-way ANOVA test, the effects of bonding type and AA on microtensile bond strength were determined.

The intragroup differences were determined using Tukey and T-tests. To analyze the microleakage scores, the Kruskal–Wallis nonparametric test was performed. All tests were conducted at α = 0.5 significance level.

The study was approved by the Research Ethics Committees of Islamic Azad University- Dental Branch Tehran - Iran (IR.IAU.DENTAL.REC.1401.053). 

## Results

The results of the two-way ANOVA test showed that the interaction between the type of universal adhesive and AA has a significant effect on the microtensile bond strength (*p*= 0.003).

**Figure 2:**
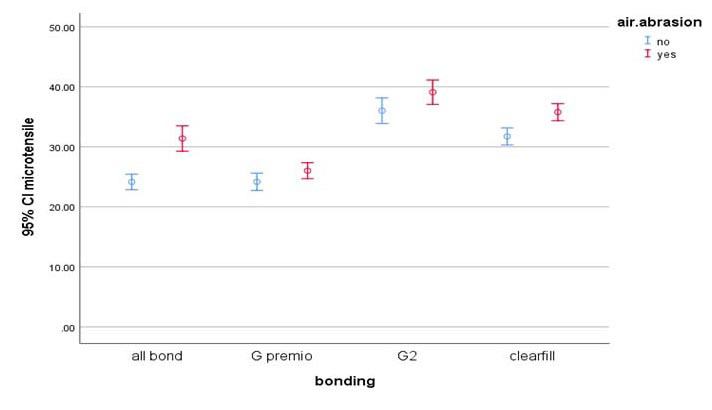
The effect of type of universal adhesive on the bond strength

T-Test was used to analyze the effect of AA on the bond strength (Table 2). The average bond strength of AL in the group with AA (37.31 MPa) was significantly higher than the group without AA (24.13 MPa) (*p*< 0.001). The average bond strength of GP bond in the group with AA (26.2 MPa) was significantly higher than the group without AA (24.16 MPa) (*p*= 0.041). The average bond strength of G2 in the group with AA (39.09 MPa) was significantly higher than the group without AA (36.01 MPa) (*p*= 0.027). The average bond strength of CSE in the group with AA (35.77 MPa) was significantly higher than the group without AA (31.72 MPa) (*p*< 0.001). The Tukey test was used to analyze the effect of type of universal adhesive on the bond strength (Figure 2). In the groups without AA, the average bond strength of G2 was significantly higher than AL (*p*< 0.001), GP (*p*< 0.001) and CSE (*p*= 0.001). In the groups with AA, the average bond strength of G2 was significantly higher than AL (*p*< 0.001), GP (*p*< 0.001) and CSE (*p*= 0.019). The distribution of failure modes in each of the studied groups is given in Table 3 according to the results; most of the failures in the groups without AA were adhesive, while the number of adhesive failures decreased in the groups with AA. Most of the failures with AA occurred in two patterns: cohesive and mix. The results of the Kruskal-Wallis test showed that there was no significant difference between the amount of microleakage in the groups with and without AA (*p*= 0.32) (Table 4). 

**Table 2:** Microtensile Bond Strength (MPa) values (means and standard deviations) of universal adhesives tested

With Air Abrasion	Without Air Abrasion	Universal Adhesive
31.37(±2.53)	24.13(±1.55)	All bond universal
26.02(±1.59)	24.16(±1.71)	G-Premio bond
39.09(±2.42)	36.01(±2.55)	G2 bond universal
35.77(±1.69)	31.72(±1.69)	Clearfill SE universal bond

**Table 3:** Number and percentage of specimens (%) according to the fracture pattern mode

Universal Adhesive	Air Abrasion	Fracture pattern		
		A	C	M
All bond universal	With	3(37.5)	2(25)	3(37.5)
	With out	4(50)	2(25)	2(25)
G-Premio bond	With	4(50)	2(25)	2(25)
	With out	4(50)	1(12.5)	3(37.5)
G2 bond universal	With	2(25)	3(37.5)	3(37.5)
	With out	3(37.5)	2(25)	3(37.5)
Clearfill SE universal bond	With	3(37.5)	2(25)	3(37.5)
	With out	4(50)	2(25)	2(25)

*Abbreviations: A: adhesive fracture mode; C: cohesive fracture mode; M: mixed fracture

**Table 4:** Frequency of microleakage scores

Universal adhesive	Air Abrasion	Microleakage score				
		0.00	1.00	2.00	3.00	4.00
All bond universal	With	10 (83.3%)	1 (8.3%)	1 (8.3%)	0 (0.0%)	0 (0.0%)
	Without	8 (66.7%)	2 (16.7%)	1 (8.3%)	1 (8.3%)	0 (0.0%)
G-Premio bond	With	7 (58.3%)	2 (16.7%)	1 (8.3%)	1 (8.3%)	1 (8.3%)
	Without	7 (58.3%)	1 (8.3%)	2 (16.7%)	1 (8.3%)	1 (8.3%)
G2 bond universal	With	11 (91.7%)	1 (8.3%)	0 (0.0%)	0 (0.0%)	0 (0.0%)
	Without	10 (83.3%)	2 (16.7%)	0 (0.0%)	0 (0.0%)	0 (0.0%)
Clearfill SE universal bond	With	9 (75%)	2 (16.7%)	1 (8.3%)	0 (0.0%)	0 (0.0%)
	Without	8 (66.7%)	2 (16.7%)	1 (8.3%)	1 (8.3%)	0 (0.0%)

## Discussion

Universal dental adhesives were introduced as versatile, multifunctional systems with reduced application steps. They are compatible with all dental hard tissue treatment modalities and capable of bonding to various restorative materials when combined with appropriate surface treatments [ 19 - 20 ]. All the tested commercial products contained the 10-MDP adhesive monomer, which has a well-documented bonding capacity with dentin [ 21 - 22 ].

In the present study, we evaluated the bond strength of the universal adhesives to dentin exclusively in the self-etch mode. This approach was chosen because self-etch is not inferior to the etch-and-rinse technique in terms of bond strength values [ 11 ]; and it provides more durable bonding after extended water ageing due to reduced degradation of the resin-infiltrated collagen [ 23 ].

As previously demonstrated, dentin treated with the total-etch bonding technique is highly susceptible to enzymatic and hydrolytic degradation. However, the dentin is partially etched by self etch bonding and some amounts of hydroxyapatite are left around the collagen grid. Therefore, with a combination affinity for hydroxyapatite, ionic bonds are created by functional monomers (10MDP) in bonding, leading to bond stability [ 24 - 25 ].

The bonding performance of Scotch bond universal was evaluated by Silva *et al*. [ 26 ] concluding no differences in bond strength in terms of AA when using this adhesive in self-etch mode. According to the literature, the abrasion of dentin with aluminum oxide causes no interference with bond strength both in self-etch mode [ 27 ]. However, in the present work, we found that pretreatment of dentinal surface utilizing AA increases the bond strength in all examined universal adhesives. The difference may be related to the versatility in acidity and composition of universal adhesives.

In the present study, dentinal surface pretreatment with AA increased microtensile bond strength for all examined universal adhesives. The dentinal bond strength was improved by pretreatment of dentin with aluminum oxide AA owing to the contact between the adhesive and dentin and incremented surface roughness [ 27 ]. Moreover, the resin monomers infiltration into the dentin increased by the superficial removal of smear layer by AA and thus increasing adhesion [ 5 ]. The increased bond strength may be also caused by the changes the dentinal surface energy resulting from abrasion with aluminum oxide. Hence, better interactions were promoted between forces of adhesion and cohesion determining the occurrence of wetting (the spreading of a liquid over a surface), and incrementing area accessible for adhesion [ 28 ].

AL revealed the most considerable increment in bond strength followed by dentin pretreatment utilizing AA, after CSE. This may be related to the universal adhesives pH. AL had the least acidity level among the adhesives studied in this work, with the pH of about 3.1 after CSE exhibiting pH of almost 2.3. Van Meerbeek *et al*. [ 9 ] investigated the bonding performance of CSE regarding AA and concluded that the µTBS of CSE to air-abraded dentin was significantly greater than all other experimental groups, which is in line with our findings. At dentin, only the diamondbur preparation prevented enough micromechanical bonding through hybridization. This is expected as the applied regular-grit diamond led to a relatively thick smear layer [ 9 ]. Such bur-based bonding effectiveness has been repeatedly reported for “mild” self-etch adhesives [ 29 - 30 ]. Despite the former case, the highest level of acidity is represented by GP among the adhesives studied in this work with the pH of about 1.5. Hence, this adhesive showed the lowest increment in bond strength after AA. As seen, “robust” self-etch and etch and rinse adhesives are related to their higher etching aggressiveness with no sensitivity to the tooth surface preparation mode [ 29 ].

The effects of thermocycling and AA on shear bond strength to dentin for self-etch adhesives were evaluated by Freeman *et al*. [ 28 ]. They reported that AA increased the mean shear bond strength to dentin, although, it was insignificant. In line with our results, for air abraded samples, after thermocycling, shear bond strength values was higher [ 28 ]. 

Dental adhesion aims at obtaining intimate adaptation of the restorative material to tooth structure [ 6 ]. However, the higher organic and water content of dentin make challenges for bonding [ 31 ]. A physical barrier is created by smear layer formed during cavity preparation, which must be dissolved or permeable. Hence, the dentin surface can be contacted with adhesive monomers [ 6 ]. The smear layer's basic composition is hydroxyapatite and changed collagen [ 32 ]. Moreover, the smear layer's morphology is different with the type of instrument creating it and the formation site [ 6 ]. The efficacy and penetration of the etch component in an adhesive system may be potentiated by decreasing the smear layer's thickness and changing the dentin surface structure after AA. Therefore, etching causes to remove a thin smear layer occluding the dentin tubule easily. Bond strength and resin tag formation are enhanced for all adhesive systems, with a higher effect for self-etch adhesives [ 28 ].

França *et al*. [ 47 ] evaluated the effects of long-term storage and AA on the bond strength of self-etch adhesives to dentin. They concluded that former dentinal AA with aluminum oxide had no effect on the bond strength means of adhesive systems at various assessment times, excepting the CSE adhesive system. Followed by three months of storage, greater mean bond strength to dentin was obtained when utilizing with aluminum oxide AA. In the current work, higher microtensile bond strength was revealed by all universal adhesives after 1,000 thermal cycles. In other words, after thermal cycles, the satisfactory impacts of AA were remained on the bond strength of universal adhesives. It was revealed that the fracture mode was influenced by AA. Using AA reduced the number of adhesive failures excluding GP. The reduced adhesive failures were dominant for AL. The data extracted from microtensile bond strength test were confirmed by these results.

According to Anja *et al*. [ 27 ], microtensile bond strength in dentin was not enhanced or impaired using AA with one-step self-etch adhesive. This is partially in line with our results.

Soares *et al*. [ 33 ] indicated that the bond strength to bovine dentin was reduced by aluminum oxide sandblasting process inconsistent with our findings. Various samples used in the studies can explain differences in the findings. While Soares *et al*. [ 33 ] performed bond strength tests on bovine teeth, our study utilized human teeth. According to Schilke *et al*. [ 34 ], the density of dentin tubules is higher significantly in human dentin compared to bovine dentin, which could clarify various findings. Furthermore, variations in adhesive bond strength measurement may result from differences in the relative proportions of inter-tubular and intratubular dentine [ 35 ], as well as the characteristics of the inter-tubular matrix between bovine and human teeth [ 36 ].

Based on scanning electron microscope (SEM) observations in former studies, aluminum oxide AA can create roughened surface, thus incrementing the surface area accessible for bonding and wetting by the adhesive resin [ 37 ].

Desired esthetic and physical properties have been obtained by the recent advances. However, their polymerization shrinkage and related stress are among the most important complications. The main factor influencing longevity is microleakage at the interface between tooth and dental restoration where restorative margins can be colored or cause incremented sensitivity in the restored tooth, secondary caries, and also pulp pathological injury. The clinical prognosis of restorations is assessed by marginal quality [ 38 ].

In the present work, we found that the quantity of microleakage in universal adhesives was not affected by AA. Thus, AA caused superficial maceration of the collagen fibers on the dentin surface and increased the hybrid layer separation from resin penetrating tubules. Therefore, the superficial structure of the dentin was weakened thus affecting the hybrid layer quality [ 39 ] leading to the creation of defects like clefts and voids. Moreover, a thin smear layer covers the dentin surface made by airborne-particle abrasion. The activity of the conditioning agent is potentiated by a thinner smear layer coating a macerated dentin surface while etching the fragile dentin surface [ 40 ]. Besides, tag formation may be hindered by failure to eliminate the smear layer forming a plug at the tubule opening [ 41 ]. It is also indicated that the rounded margins created by AA help reduce marginal microleakage and polymerization stress [ 42 ]. Our results may be explained by these conflicting effects.

The effects of AA and thermocycling on resin adaptation to dentin were evaluated by Freeman *et al*. [ 28 ] for self-etch adhesives. They reported that AA and thermocycling compromised the adaptation to dentin. Despite increasing the number, diameter, and length of resin tags, AA was associated with a higher number of defects in the hybrid layer on the dentin surface [ 28 ].

For optimum dentin bonding, the demineralized dentin tubule must be penetrated by the adhesive before polymerization [ 43 ]. Nevertheless, the adhesive may be separated from the tubule wall by polymerization shrinkage thus producing hollow resin tags [ 44 ]. Furthermore, the formation of resin tags may not contribute to bond strength, as separation of the hybrid layer from the adjacent dentin can occur following polymerization shrinkage and restoration aging.

In the current study, AA did not affect the amount of microleakage but did increase the micro-tensile bond strength of all universal adhesives. It is important to note that during composite polymerization, the adhesive layer in cavity preparations is subjected to shrinkage stresses, which are absent in microtensile bond strength specimens. Therefore, the loss of adaptation was not explained by bond strength results.

Atalay *et al*. [ 45 ] evaluated the microleakage of a Universal adhesive bond in Class V resin composite restorations using Er,Cr:YSGG laser surface treatment. Their results showed that laser etching had no significant effect on the microleakage at dentin margins. Atalay *et al*. [ 45 ] also evaluated the universal adhesive bond using the self-etch approach, similar to our study. The findings from their study are in agreement with the data obtained from our microleakage tests.

The effects of surface modification of dentin were explored by Almojaly *et al*. [ 41 ] utilizing Er, Cr: YSGG phototherapy on microleakage scores. They found higher microleakage scores in laser treated groups caused by high power density, heat damage, and dentin crystals denaturation.

Microleakage related to composite restorations was compared by Arora *et al*. [ 46 ] in Class V cavities preconditioned with AA for Adper Single bond. It was indicated that microleakage was less predominant in teeth, for which AA was utilized for preconditioning the cavity. However, differences in adhesive types across studies could explain varying results. Thus, the effect of AA on microleakage may depend on the specific bonding system used, and further research is needed to confirm its generalizability. 

As we know, the effect of AA on the bonding performance of universal adhesives was not studied so far. Considering the extensive spread of universal adhesives in clinical practice, the present work can be used to enhance these adhesives' performance. Thus, further studies are required to assess other features of these adhesives using AA.

The findings of this study should be interpreted in light of its limitations, particularly its *in vitro* design, which may not fully replicate the complex oral environment, including factors such as moisture, temperature fluctuations, masticatory forces, and long-term degradation. Therefore, further clinical studies are necessary to validate the long-term effectiveness of air abrasion pretreatment in enhancing the performance of universal adhesives.

## Conclusion

Within the limitation of this *in vitro* study it can be concluded that pretreatment of dentin with AA increases the bond strength and durability of the universal adhesives and this effect is greater in universal adhesives with higher pH. Moreover, pretreatment of dentin with AA does not affect the microleakage of universal adhesives.

## References

[1] Salz U, Bock T ( 2010). Testing adhesion of direct restoratives to dental hard tissue- a review. J Adhes Dent.

[2] Mjor IA, Gordan VV ( 2002). Failure, repair, refurbishing and longevity of restorations. Oper Dent.

[3] De Munck J, Van Landuyt K, Peumans M, Poitevin A, Lambrechts P, Braem M, et al ( 2005). A critical review of the durability of adhesion to tooth tissue: methods and results. J Dent Res.

[4] Estafan D, Agosta C ( 2003). Eliminating microleakage from the composite resin system. Gen Dent.

[5] Haller B ( 2000). Recent developments in dentin bonding. Am J Dent.

[6] Perdigao J ( 2007). New developments in dental adhesion. Dent Clin North Am.

[7] Harashima T, Kinoshita J, Kimura Y, Brugnera A, Zanin F, Pecora JD, et al ( 2005). Morphological comparative study on ablation of dental hard tissues at cavity preparation by Er:YAG and Er,Cr:YSGG lasers. Photomed Laser Surg.

[8] Brkanović S, Sever EK, Vukelja J, Ivica A, Miletić I, Krmek SJ ( 2023). Comparison of different universal adhesive systems on dentin bond strength. Materials.

[9] Van Meerbeek B, De Munck J, Mattar D, Van Landuyt K, Lambrechts P ( 2003). Microtensile bond strengths of an etch & rinse and self-etch adhesive to enamel and dentin as a function of surface treatment. Oper Dent.

[10] Gray GB, Carey GP, Jagger DC ( 2006). An in vitro investigation of a comparison of bond strengths of composite to etched and air-abraded human enamel surfaces. J Prosthodont.

[11] Rosa WL, Piva E, Silva AF ( 2015). Bond strength of universal adhesives: A systematic review and meta-analysis. J Dent.

[12] Santander-Rengifo F, Carreras-Presas CM, Aroste-Andía R, Hernández-Huamaní E, Gavilán-Chávez P, Cervantes-Ganoza L, et al ( 2024). Microtensile Bond Strength and Failure Mode of Different Universal Adhesives on Human Dentin. Int Dent J.

[13] Wang R, Shi Y, Li T, Pan Y, Cui Y, Xia W ( 2017). Adhesive interfacial characteristics and the related bonding performance of four self-etching adhesives with different functional monomers applied to dentin. J Dent.

[14] Carrilho E, Cardoso M, Marques Ferreira M, Marto CM, Paula A, Coelho AS ( 2019). 10-MDP based dental adhesives: Adhesive interface characterization and adhesive stability: A systematic review. Materials (Basel).

[15] Jang JH, Lee MG, Woo SU, Lee CO, Yi JK, Kim DS ( 2016). Comparative study of the dentin bond strength of a new universal adhesive. Dent Mater J.

[16] Rafael CF, Quinelato V, Morsch CS, DeDeus G, Reis CM ( 2016). Morphological analysis of dentin surface after conditioning with two different methods: Chemical and mechanical. J Contemp Dent Pract.

[17] D'Amario M, Piccioni C, Di Carlo S, De Angelis F, Caruso S, Capogreco M ( 2017). Effect of airborne particle abrasion on microtensile bond strength of total-etch adhesives to human dentin. Biomed Res Int.

[18] International standards organization Dental materials- Testing of adhesion to tooth structure. Geneva: The organization. 2003; ISO/TS 11405. https://www.iso.org/standard/31486.html.

[19] Nagarkar S, Theis-Mahon N, Perdigao J ( 2019). Universal dental adhesives: Current status, laboratory testing, and clinical performance. J Biomed Mater Res B Appl Biomater.

[20] Lumkemann N, Eichberger M, Stawarczyk B ( 2019). Different surface modifications combined with universal adhesives: the impact on the bonding properties of zirconia to composite resin cement. Clin Oral Investig.

[21] Yoshida Y, Nagakane K, Fukuda R, Nakayama Y, Okazaki M, Shintani H, et al ( 2004). Comparative study on adhesive performance of functional monomers. J Dent Res.

[22] Hardan L, Bourgi R, Kharouf N, Mancino D, Zarow M, Jakubowicz N, et al ( 2021). Bond strength of universal adhesives to dentin: A systematic review and meta-analysis. Polymers.

[23] Zhang ZY, Tian FC, Niu LN, Ochala K, Chen C, Fu BP, et al (2016). Defying ageing: An expectation for dentine bonding with universal adhesives?. J Dent.

[24] Breschi L, Mazzoni A, Ruggeri A, Cadenaro M, Di Lenarda  R, De Stefano Dorigo  E ( 2008). Dental adhesion review: aging and stability of the bonded interface. Dent Mater.

[25] Armstrong SR, Jessop JL, Vargas MA, Zou Y, Qian F, Campbell JA, et al ( 2006). Effects of exogenous collagenase and cholesterol esterase on the durability of the resin-dentin bond. J Adhes Dent.

[26] Sutil B, Susin AH ( 2017). Dentin pretreatment and adhesive temperature as affecting factors on bond strength of a universal adhesive system. J Appl Oral Sci.

[27] Anja B, Walter D, Nicoletta C, Marco F, Pezelj Ribaric S, Ivana M ( 2015). Influence of air abrasion and sonic technique on microtensile bond strength of one-step self-etch adhesive on human dentin. Sci World J.

[28] Freeman R, Varanasi S, Meyers IA, Symons AL ( 2012). Effect of air abrasion and thermocycling on resin adaptation and shear bond strength to dentin for an etch-and-rinse and self-etch resin adhesive. Dent Mater J.

[29] Inoue H, Inoue S, Uno S, Takahashi A, Koase K, Sano H ( 2001). Microtensile bond strength of two single-step adhesive systems to bur-prepared dentin. J Adhes Dent.

[30] Van Meerbeek B, Vargas M, Inoue S, Yoshida Y, Peumans M, Lambrechts P, et al ( 2001). Adhesives and cements to promote preservation dentistry. Oper Dent.

[31] Pashley DH ( 1992). The effects of acid etching on the pulpodentin complex. Oper Dent.

[32] Eick JD, Cobb CM, Chappell RP, Spencer P, Robinson SJ ( Quint Int 1991). The dentinal surface: its influence on dentinal adhesion. Part I.

[33] Soares CJ, Castro CG, Santos Filho  PC, da Mota AS ( 2007). Effect of previous treatments on bond strength of two self-etching adhesive systems to dental substrate. J Adhes Dent.

[34] Schilke R, Lisson JA, Bauss O, Geurtsen W ( 2000). Comparison of the number and diameter of dentinal tubules in human and bovine dentine by scanning electron microscopic investigation. Arch Oral Biol.

[35] Hirayama A, Yamada M, Miake K ( 1986). An electron microscopy study on dentinal tubules of human deciduous teeth. Shikwa Gakuho.

[36] Nor JE, Feigal RJ, Dennison JB, Edwards CA ( 1996). Dentin bonding: SEM comparison of the resin-dentin interface in primary and permanent teeth. J Dent Res.

[37] Nikaido T, Kataumi M, Burrow MF, Inokoshi S, Yamada T, Takatsu T ( 1996). Bond strengths of resin to enamel and dentin treated with low-pressure air abrasion. Oper Dent.

[38] Taylor MJ, Lynch E ( 1992). Microleakage. J Dent.

[39] Nikaido T, Yamada T, Koh Y, Burrow MF, Takatsu T ( 1995). Effect of air-powder polishing on adhesion of bonding systems to tooth substrates. Dent Mater.

[40] Pashley DH, Carvalho RM ( 1997). Dentine permeability and dentine adhesion. J Dent.

[41] Almojaly SA, Sulaiman Al-Hamdan  R, Alrahlah A, Qutub OA, Alnajashi S, Vohra F, et al ( 2019). Effect of Er, Cr: YSGG on bond strength and microleakage of dentin bonded to resin composite with different distance and irradiation time. Photodiagnosis Photodyn Ther.

[42] Arias VG, Campos IT, Pimenta LA ( 2004). Microleakage study of three adhesive systems. Braz Dent J.

[43] Giachetti L, Bertini F, Scaminaci Russo  D ( 2004). Investigation into the nature of dentin resin tags: a scanning electron microscopic morphological analysis of demineralized bonded dentin. J Prosthet Dent.

[44] Tay FR, Gwinnett AJ, Pang KM, Wei SH ( 1994). Structural evidence of a sealed tissue interface with a total-etch wet-bonding technique in vivo. J Dent Res.

[45] Atalay C, Uslu A, Yazici AR ( 2021). Does laser etching have an effect on application mode of a universal adhesive?-A microleakage and scanning electron microscopy evaluation. Microsc Res Tech.

[46] Arora A, Acharya SR, Vidya SM, Sharma P ( 2012). A comparative evaluation of dentinal hypersensitivity and microleakage associated with composite restorations in cavities preconditioned with air abrasion: An ex vivo study. Contemp Clin Dent.

[47] França FM, dos Santos  AJ, Lovadino JR ( 2007). Influence of air abrasion and long-term storage on the bond strength of self-etching adhesives to dentin. Oper Dent.

